# Comparison of two soft tissue substitutes for the treatment of gingival recession defects: an animal histological study

**DOI:** 10.1590/1678-7757-2018-0584

**Published:** 2019-10-07

**Authors:** Fernando Suárez-López Del Amo, Juan C. Rodriguez, Farah Asa'ad, Hom-Lay Wang

**Affiliations:** 1 University of Oklahoma Health Sciences Center, Department of Periodontics, Oklahoma City, OK, USA; 2 University of Michigan School of Dentistry, Department of Periodontics and Oral Medicine, Ann Arbor, MI, USA; 3 The Sahlgrenska Academy at the University of Gothenburg, Institute of Odontology, Göteborg, Sweden

**Keywords:** Gingival recession, Esthetics, Dental, Surgery, Plastic, Treatment outcome

## Abstract

**Objectives:**

This study aimed to compare two different soft tissue replacement grafts in their ability to treat gingival recession defects and successfully integrate with the surrounding tissues.

**Methodology:**

Nine beagle dogs were included and followed up to 10 weeks. Sites for intervention were allocated to one of the grafting materials investigated. Treatment consisted of coronally advanced flap combined with one of the two soft tissue substitutes on a previous surgically created defect. Materials employed were porcine-derived acellular dermal matrix (ADM) [Novomatrix^™^ (Test)] and collagen-based matrix (CBM) [Mucograft^®^ (Control)]. Animals were sacrificed at 2, 6, and 10 weeks postoperatively and compared using descriptive histology and histomorphometric outcomes.

**Results:**

Macroscopic findings were similar between test and control groups at all intervals. After 10 weeks, both groups demonstrated successful incorporation of the grafting materials without signs of rejection and with comparable tissue integration. The histomorphometric data were similar between groups at 2 weeks; however, the test group provided greater root coverage and increase in tissue thickness than the control at 6- and 10-weeks post surgically.

**Conclusions:**

Both porcine-derived ADM and CBM revealed similar histological outcomes with successful integration and absence of adverse events. Test group provided superior outcomes regarding root coverage and increase in tissue thickness.

## Introduction

Soft tissue augmentation procedures have been extensively employed for the correction of mucogingival deformities, especially soft tissue recession defects. This condition affects a large portion of the population regardless of the oral hygiene standards;^1^ the estimation is that 23.8 million people (22.5%) in the United States over 29 years old present at least one tooth surface with ≥3mm gingival recession (GR).^2^ The extent, prevalence, and severity of these mucogingival deformities increase with age^2^ and continue^3^ to progress with the apical displacement of the gingival marginal, if left untreated. GR is also associated with a series of undesirable consequences including esthetic disharmony, hypersensitivity, and root caries. Consequently, the prevention and treatment of GR defects remain as the focus in the periodontal plastic surgery investigations. As such, multiple approaches have been employed and investigated for the correction of GR including, but not limited to; guided tissue regeneration (GTR),^4^ coronally advanced flap (CAF),^5^ tunneling techniques,^6^ and lateral pedicle flap (LPF).^7^ These techniques have been used alone or combined with different auto-, allo-, and xenografts.^8^

To date, the use of autogenous connective tissue graft (CTG) has no equivalent regarding its properties, predictability, and long-term outcomes^8^ (8). Although CTG is considered the gold standard, the use of alternative grafting materials presents several advantages, namely reduced morbidity, reduced surgical time, and unlimited availability. Hence, investigators continue to seek non-autogenous soft tissue grafting substitutes and other alternative methods. Among these are: acellular dermal matrix (ADM),^9^ porcine collagen matrix,^10^,^11^ enamel matrix derivatives,^12^ and GTR.^4^

ADM represents one of the most widely used substitutes for autogenous connective tissue. Since its introduction in the 1990s, numerous studies have investigated the use of ADM in periodontal plastic surgery procedures.^9^,^13^,^14^ Increase in root coverage and in tissue thickness, and augmentation of the keratinized tissue have been reported after treatment with ADM.^9^,^14^,^15^ In addition, it has been demonstrated that neither the orientation nor the processing technique for ADM play a role in the final outcomes.^14^,^16^ Consequently, the use of ADM is considered nowadays a predictable alternative for the treatment of GR defects.

The success of periodontal plastic surgery relies primarily on the resolution of the GR defect and on several other parameters including: reduction in probing depth; increase in tissue thickness; keratinized tissue gain; and the overall aesthetic outcomes. However, although these clinical parameters have been extensively investigated with numerous different techniques and grafting materials, studies reporting the histological outcomes are still limited.^17^ Nevertheless, adequate integration and adaptation of grafting materials to the surrounding tissues and against the denuded root surface is also important due to its role in the long-term stability.

Within the few investigations reporting the histological outcomes, the employment of CTG and biological agents for the treatment of GR appears to be evaluated more extensively.^18^–^2^. Diversely, the histological outcomes of ADM, more specifically of porcine-derived ADM, remain to be investigated. Hence, this investigation aimed to evaluate and histologically compare two different porcine-derived soft tissue substitutes: [Collagen-based matrix (CBM) {Mucograft^®^ (Control)} and ADM {NovoMatrix^™^ (Test)}] combined with CAF for the treatment of GR defects. The primary objective of this investigation was to evaluate the safety and tissue response of the new porcine-derived ADM. Secondary objectives include histomorphometric analysis and comparison between both materials investigated.

## Methodology

### Study design

This investigation was conducted on 9 beagle dogs (*Canis familiaris*), with an age range from 16 – 36 months. Four sites per animal (maxillary and mandibular canines) were included and allocated to one of the grafting materials. As such, a total of 36 sites were surgically treated, 18 as control and 18 as test. Three animals were sacrificed at each interval (at 2, 6, and 10 weeks postoperatively) providing a total of 12 (6 test and 6 control) sites for evaluation. Approval by the NAMSA Northwood Division Institutional Animal Care and Use Committee (IACUC) was obtained before conducting the study. The sites were predetermined to ensure equal and equivalent distribution of sites and material among the animals included. Soft tissue thickness and coverage, local tissue reaction, and tissue integration were evaluated following the treatment of previously created mucogingival recession defects with two different soft tissue substitutes combined with CAF. Each animal received two porcine-derived materials {test (NovoMatrix^™^ LifeCell, California, USA) and two control (Mucograft, Geistlich Pharma AG, Wolhusen, Switzerland)}.

### Sedation, anesthesia, and surgical procedure

At inclusion, animals weighed from 7.6 kg to 10.9 kg. A minimum of 7 days was required for acclimatization of the animals. Tattoos were used as identification method. Animals were fasted the day prior. On the day of surgery, tramadol (2-5 mg/kg, by mouth (PO)) was given before premedication for the procedure. Each animal was pre-anesthetized with an intramuscular dose of acepromazine maleate (0.2 mg/kg). An intravenous catheter was placed in a forelimb for intravenous access and a saline drip for hydration. General anesthesia was induced by intravenous propofol (4 mg/kg, to effect). A non-medicated ophthalmic ointment was applied to both eyes of each animal to protect their corneas from drying. Each animal was intubated and placed on isoflurane inhalant anesthetic for continued general anesthesia. In addition, the animals were maintained on a supplemental heating source and their vital signs (temperature, heart rate, respiration rate, oxygen saturation levels) were monitored during the procedure. Postoperatively, an additional dose of tramadol (2-5 mg/kg, PO) was given at the end of the surgical day and then twice daily on days 1 through 4. Clindamycin (7-11 mg/kg, PO) was administered once daily on days 0 (postoperative) through 7.

**Figure 1:**
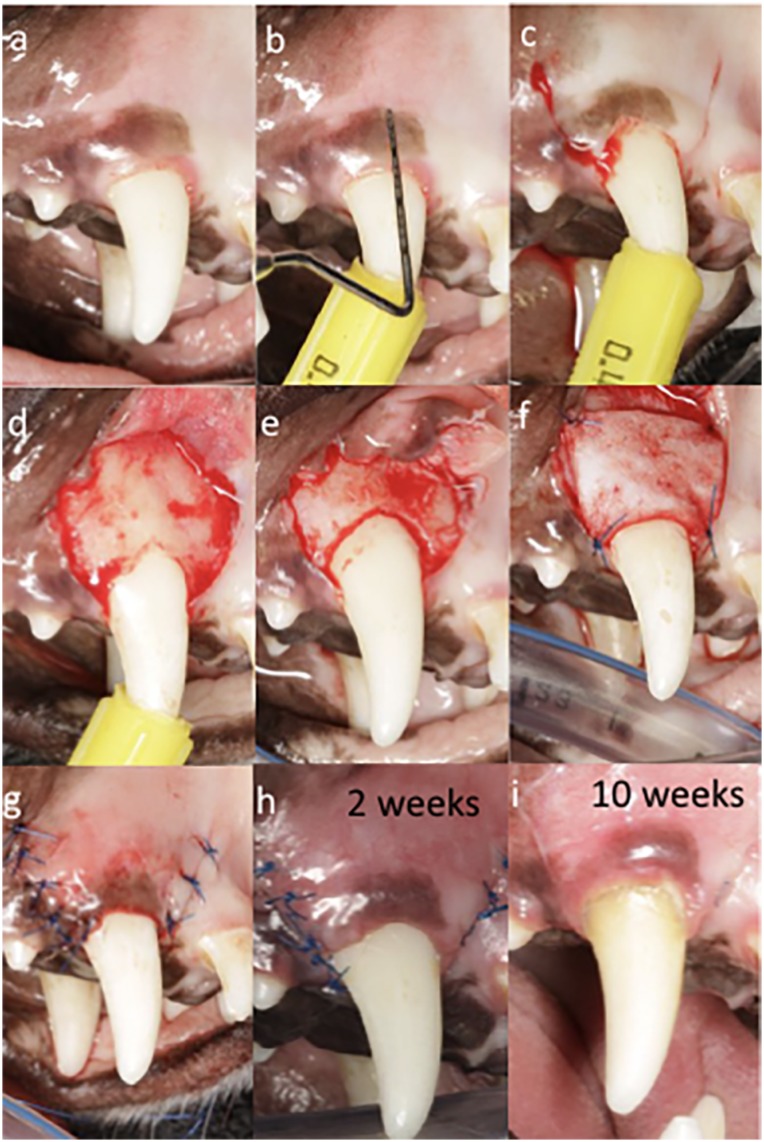
Surgical procedure for test group. A: Preoperative view after manual instrumentation. B: Probe for identification of 3 mm of tissue collar to be removed. C: Operative view after excision of 3 mm of tissue collar. Coronal notch performed at the level of the new surgically created gingival margin and vertical incisions. D: Flap opening. E: Ostectomy to remove 3 mm from first notch and creation of second notch. F: Adaptation and suturing of test device. G: Immediate postoperative view. H: Two weeks follow-up. I: Ten weeks follow-up

**Figure 2:**
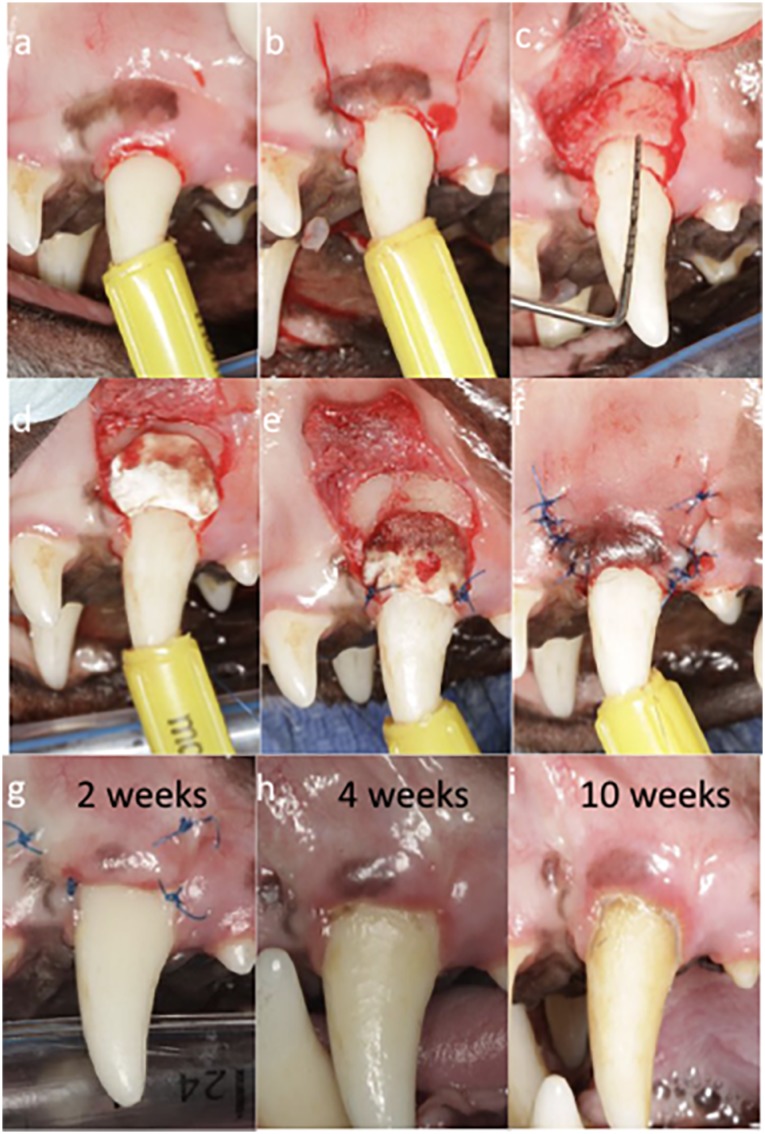
Surgical procedure for control group. A: Preoperative view after manual instrumentation. B: Operative view after excision of 3 mm of tissue collar. Coronal notch performed at the level of the new surgically created gingival margin and vertical incisions. C: Operative view after flap opening and ostectomy performed demonstrating 3 mm from the first to the second notch. D: Adaptation of control device. E: Suturing of control device F: Immediate postoperative view. G: Two weeks follow-up. H: Four weeks follow-up I: Ten weeks follow-up

Before surgery, all teeth included were carefully debrided by manual instrumentation (Figure 1a). Later, recession defects were created by surgical excision of 3 mm of keratinized mucosa on the buccal aspects of the sites (Figure 1b). Intrasulcular incisions connecting with two vertical incisions at mesial and distal line angles were created at the buccal side with a 15C blade. Both vertical incisions extended apically beyond the mucogingival junction (MGJ) in an oblique direction. Then, small round bur was used to create the first notch (Figure 1c). Full-thickness flap was reflected slightly apical to the MGJ followed by partial-thickness flap to obtain flap mobility (Figure 1d). Ostectomy was later performed for removal of bone from a distance of 3 mm apical from the previously created notch. Rotatory instrument with end-cutting bur was employed for the ostectomy. Then, a second notch was created similarly following the alveolar crest (Figure 1e). After proper preparation of the grafting substitute, following the manufacturer's recommendations and adjusting the material to the size of the recipient site, it was sutured using simple interrupted sutures until stability was achieved (Figure 1f). Simple interrupted sutures ranged from 2 to 4 depending on the case. Next, the flap was coronally repositioned ensuring coverage of the grafting substitute above the most coronal notch (Figure 1g). The flap was stabilized with simple interrupted and sling sutures. When necessary, 0.5 carpule of 2% Xylocaine with 1:50K epinephrine was utilized for hemostasis during the surgery. Sutures were later removed at 2 weeks (Figure 1h), and animals were then followed up for 10 weeks (Figure 1i). A similar protocol was performed for both control and test groups (Figures 1 and 2).

### Observations and follow-up care

Animals were observed daily for general health. Body weights were measured preoperatively and weekly thereafter during the study. Following surgery, the wounds were irrigated/flushed with 0.12% chlorhexidine twice per day to keep the wounds clean. Irrigation was conducted in the morning and after the animals were fed. During the irrigation procedures, the sites of incisions were checked for patency. After the wounds had closed (healed), the irrigation was continued once a day (after eating) for one additional week. Gingival health was determined twice a week (minimum of two days apart) following surgery. The sites were evaluated for maintenance of suture line closure, edema, evidence of tissue necrosis, and evidence of infection. Once the site was deemed healed by the veterinarian, observations were conducted once a week. Any abnormalities were noted. Suture removal was conducted approximately 2 weeks after surgery. Photographs of the sites were taken every two weeks using appropriate labels and scale. After 2, 6, and 10 weeks after surgery, euthanasia was carried out by an intravenous injection of a sodium pentobarbital-based euthanasia solution. The portion of the jaw containing the defects was removed from the head. The jawbone was trimmed as needed to remove excess bone, while carefully preserving the integrity of soft tissues at and near the sites. After trimming, the defect sites were placed in 10% neutral buffered formalin (10% NBF).

### Tissue Preparation for Histological Analysis

After fixation, the defect sites were processed using EXAKT^®^. Briefly, defect sites were embedded in plastic, then sectioned, surface etched and stained. Each defect site was bisected into two sections. One slide from one section was cut and stained with hematoxylin and eosin (H&E). At least one slide was then cut from each of the two sections and stained with Masson-Goldner trichrome.

### Outcomes Evaluated

Tissue integration, tissue remodeling, and biomaterial-specific inflammatory response were evaluated histologically in the region of interest (ROI), which was represented by the area of soft tissues extending from the coronal to the apical notch. Root coverage and changes in tissue thickness were assessed histomorphometrically.

### Descriptive histology and histomorphometric outcomes

A single masked, calibrated examiner (FA) assessed all of the slides and demonstrated a pre- and post- study calibration inter- and intraexaminer error of <5% compared with a standard examiner. Tissue integration, remodeling, and biomaterial-specific inflammatory response were histologically evaluated using light microscope and computer-assisted image analysis. Briefly, regions of interest were visualized using a microscope (Nikon Eclipse TS100) at objectives: 4x and 10x for H&E and Masson-Goldner's Trichrome staining.

Then, images of histological slides in both stains were captured using a digital camera (Nikon DS-Fi2 color digital) at 0.75x and 4.0x magnifications. Images were exported to ImageJ software (National Institute of Health, USA; http://rsbweb.nih.gov/ij/) for qualitative histological analysis to evaluate tissue response and integration of both implanted biomaterials. Degradation of biomaterials was calculated by percentage as the following: surface area of the biomaterial/Surface area of the covering flap & augmented area x 100. Extension of degradation was graded as the following: slight (10-30%), moderate (>30% – 50%), and severe (>50%). Percentages of inflammatory infiltrate and blood vessels (BV) were calculated, and the presence of elastic fibers was assessed as well. Possible adverse tissue reactions such as necrosis were also identified.

**Figure 3:**
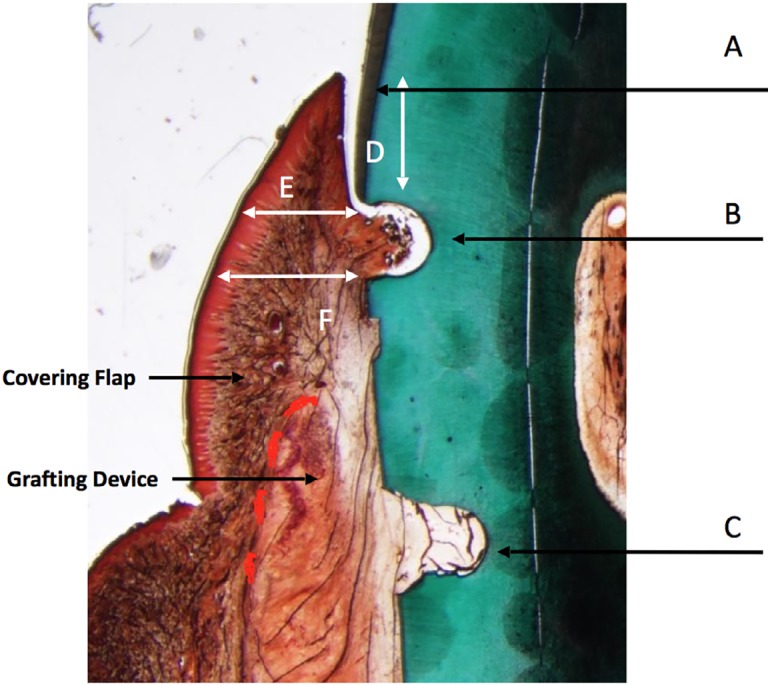
Representation of histomorphometric measurements. A: Gingival margin. B: Coronal notch. C: Apical notch. D: Gingival margin to notch distance (root coverage). E: Tissue thickness 2 mm below gingival margin. F: Tissue thickness at coronal notch

All the analyses were done in the ROI. Histomorphometric data were evaluated for root coverage and gingival tissue thickness. The histomorphometric outcomes were evaluated as shown in Figure (3); the following microscopic measurements were obtained in micrometers from the scanned or photographed images: (1) Distance from the coronal notch to the gingival margin (determination of root coverage), (2) Buccolingual tissue thickness at two points: at 2 mm below the gingival margin and at the coronal notch (Figure 3). The histomorphometric data obtained for each of the slides selected at different levels throughout the defect site were averaged to represent the entire defect area. Statistical comparisons (ANOVA) were performed to determine if significant differences were present between the test and control groups for root coverage and gingival tissue thickness. T-test was used to compare the changes between groups regarding the inflammatory cell infiltrate and blood vascularization.

## Results

### Clinical and macroscopic findings

Macroscopic findings were similar between test and control groups at all intervals. All the animals remained healthy and without systemic complications during the investigation period. One control and one test site experienced early incision line opening. Similarly, some soft tissue dehiscence and/or delayed wound healing occurred at the early stages. These phenomena seemed not to be correlated with any of the treatment groups.

### Descriptive Histology

For the control and test groups, samples were obtained and assessed at 2, 6, and 10 weeks after implantation. Parameters evaluated included histological descriptive analysis (tissue reaction; integration, and inflammatory response) and histomorphometric outcomes.

#### A) Collagen-Based Matrix (CBM):

**Figure 4:**
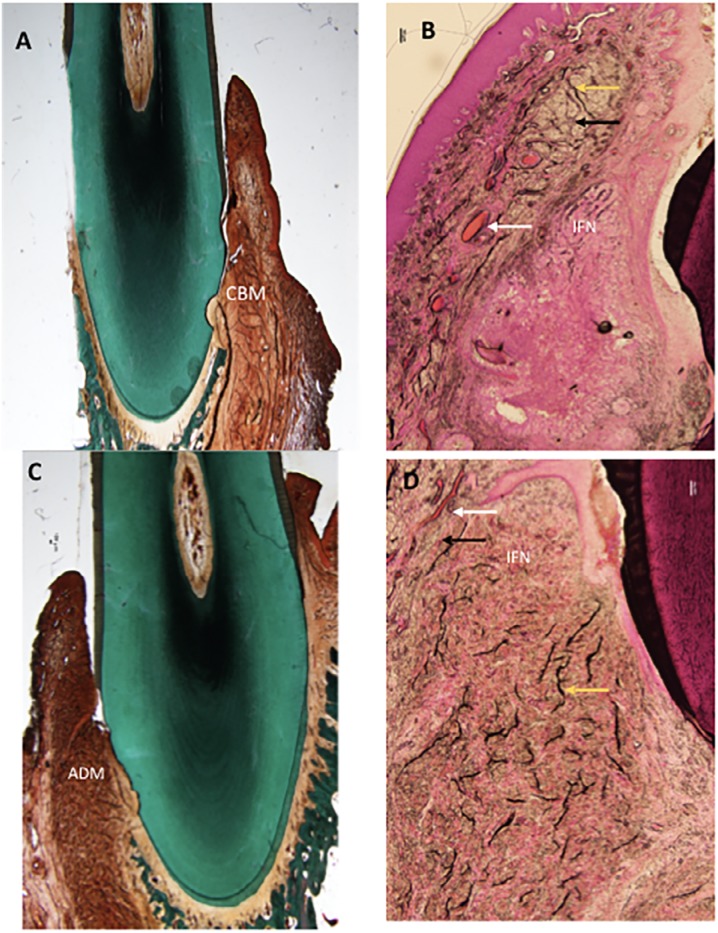
Histology slides of both groups at two weeks. A: Masson-Goldner's stained CBM group (control) at magnification (0.75x). B: H&E stained CBM group at magnification (4x) C: ADM group (test) Masson-Goldner's stained at magnification (0.75x) D: H&E stained ADM group at magnification (4x). White arrow: Blood vessels, Black arrow: Collagen fiber, Yellow arrow: Elastic fibers. IFN: Inflammatory infiltrate, CBM: Collagen-Based Matrix, ADM: Acellular Dermal Matrix

Two weeks after implantation, CBM remodeling and integration in the implantation bed were observed, showing slight degree of degradation (Figure 4A). Significant local inflammatory reaction was absent as few infiltrating neutrophils were present (Figure 4B).

**Figure 5:**
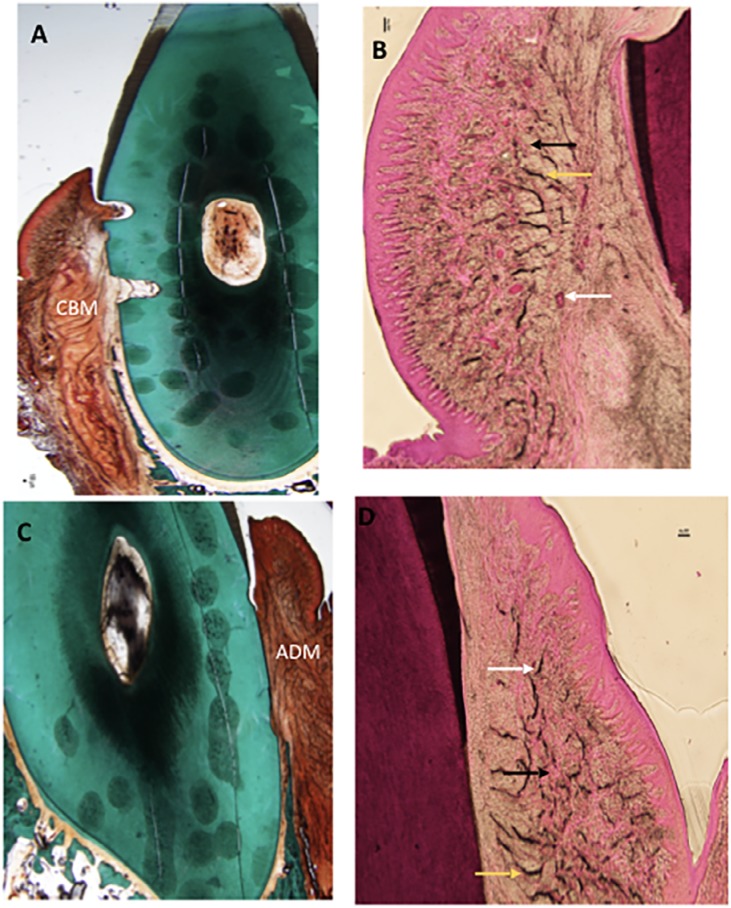
Histology slides of both groups at six weeks. A: Masson-Goldner's stained CBM group (control) at magnification (0.75x). B: H&E stained CBM group at magnification (4x) C: ADM group (test) Masson-Goldner's stained at magnification (0.75x) D: H&E stained ADM group at magnification (4x). White arrow: Blood vessels, Black arrow: Collagen fiber, Yellow arrow: Elastic fibers. CBM: Collagen-Based Matrix, ADM: Acellular Dermal Matrix

Six weeks after implantation, CBM was well integrated in the implantation bed, with a moderate degree of material degradation (Figure 5A), and signs of local inflammation were absent (Figure 5B).

**Figure 6:**
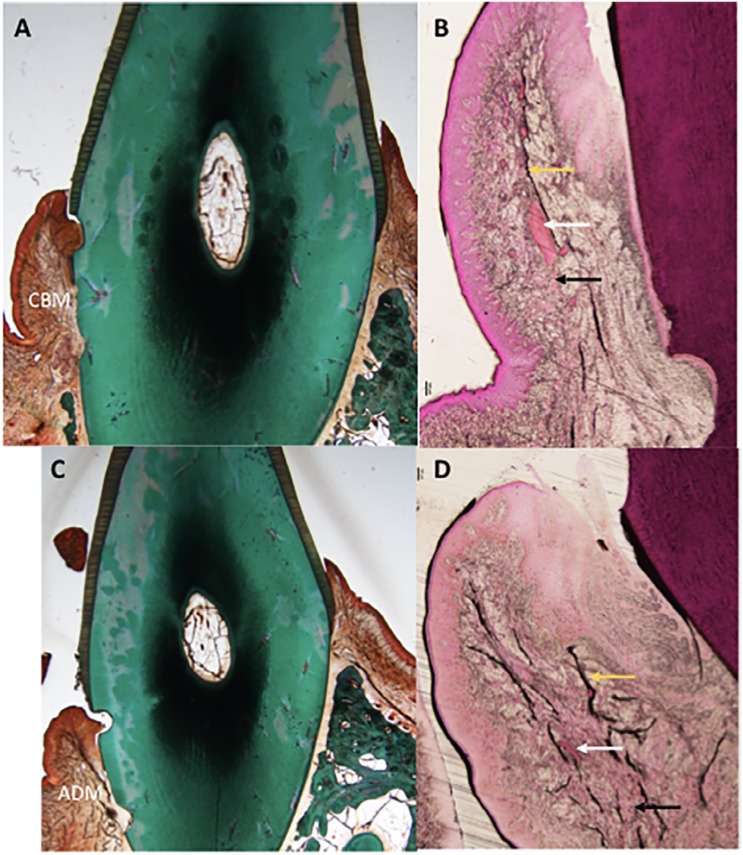
Histology slides of both groups at ten weeks. A: Masson-Goldner's stained CBM group (control) at magnification (0.75x). B: H&E stained CBM group at magnification (4x) C: ADM group (test) Masson-Goldner's stained at magnification (0.75x) D: H&E stained ADM group at magnification (4x). White arrow: Blood vessels, Black arrow: Collagen fiber, Yellow arrow: Elastic fibers. CBM: Collagen-Based Matrix, ADM: Acellular Dermal Matrix

Similar observations were reported 10 weeks after implantation as local inflammatory response was absent, and tissues were remodeled and integrated, with a moderate grade of degradation (Figure 6A and Figure 6B). Foreign body response and/or necrosis were absent at all points of time, indicating a favorable tissue reaction. Few elastic fibers were evident in the deeper regions of the grafted area at all points of time (Figure 4B, 5B, 6B).

#### B) Acellular Dermal Matrix (ADM):

Two weeks after implantation, histologic assessment showed ADM was integrated into the surrounding soft tissues, showing slight degradation (Figure 4C). Healthy soft tissues were present at the implantation bed; tissue response was favorable, and the implanted material was non-irritant as indicated by the absence of multinucleated giant cells and lymphocytes. However, only slight infiltration of neutrophils was observed (Figure 4D).

Six weeks after implantation, ADM was integrated into the surrounding tissues, being moderately degraded (Figure 5C). No signs of any local inflammation were present (Figure 5D).

Ten weeks after implantation, ADM was integrated into the surrounding tissues, being moderately degraded (Figure 6C). Further observations were similar to that reported at six weeks; local inflammatory infiltrate was absent (Figure 6D).

Foreign body response and/or necrosis were not observed at any point of time. Based on these findings, one can conclude that ADM is a nonirritant material.

Elastic fibers were evident in the deeper regions of the grafted area, during all points of time. Concentration of elastic fibers was higher during all points of time compared with the CBM group (Figures 4D, 5D, 6D).

### Histomorphometric Outcomes

**Figure 7:**
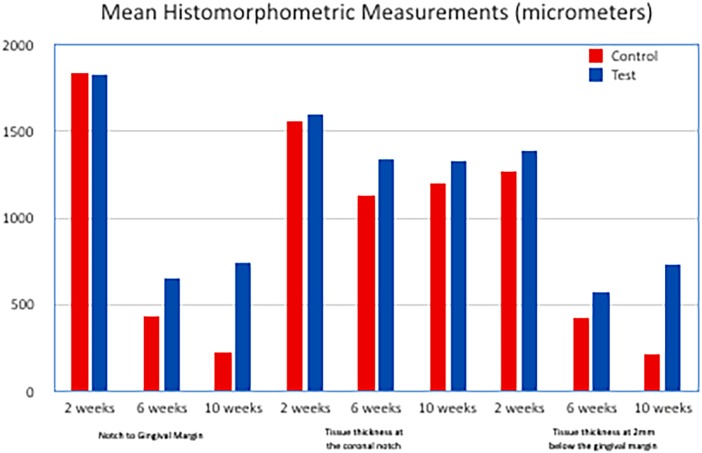
Histomorphometric results

Histomorphometric analysis was performed to evaluate the root coverage and gingival thickness of the samples studied. All data are shown in Table 1 and Figure 7. For both treatment groups, mean values for all histomorphometric measurements were higher at the 2-week time point than at the 6- or 10-week time points, indicating decreased root coverage for each treatment group after 2 weeks. The 6- and 10-week time point values were similar within each treatment group, which suggests minimal or no further loss of root coverage by 10 weeks. At the 6- and 10-week time points, the mean histomorphometric values trended higher for sites treated with ADM than for sites treated with CBM, and the trend was statistically significant (*p*<0.05) for the total mean of buccolingual tissue thickness at the coronal notch at 10 weeks. The trend toward higher values for gingival thickness and the distance from the notch to the gingival margin suggest ADM may provide more root coverage than CBM 6 and 10 weeks after implantation.

**Table 1:** Histomorphometric outcomes

Interval	Treatment group	Mean Histomorphometric Measurements (micrometers)				
	(n=6 for each group, at each point of time)	Distance from the notch to the gingival margin - all values		Tissue thickness at 2mm below the gingival margin	Tissue thickness at the coronal notch	
		All values	Positive values only	All values	All values	Positive values only
2 weeks	Control	1839.2	NA	1557.1	1272.7	NA
	Test	1826.4	NA	1595.5	1391.7	NA
6 weeks	Control	437.2	601.1	1135.2	420.9	578.8
	Test	656.7	820.8	1343.4	576.8	721
10 weeks	Control	226.4*	565.9	1197.6	217.9*	544.8
	Test	743.0*	817.3	1330	735.4*	808.9

NA= Not Applicable (section that met the required criteria not available);

* Statistically significant difference (p<0.05)

Percentage of inflammatory infiltrate and blood vessels decreased over time for the CBM group. The ADM group presented less vascularization, and less inflammatory infiltrate than the CBM group during all points of time. Inflammatory infiltrates and blood vessel percentages decreased at all time intervals, similar to the CBM group (Table 2). These changes between both groups were statistically significant only for vascularization (*p*<0.05).

**Table 2:** Mean percentages of inflammatory cell infiltrate & blood vessels in control and test groups, within different points of time

Interval	Treatment group (n=6 for each group, at each point of time)	Mean Inflammatory cell infiltrate (%± SD)	Blood Vessels (%± SD) *
2 weeks	Control	2.19 ± 1.75	1.96 ± 1.55
	Test	1.53 ± 0.73	0.96 ± 0.89
6 weeks	Control	2.03 ± 2.20	1.66 ± 1.89
	Test	0.81 ± 0.85	0.76 ± 1.11
10 weeks	Control	1.17 ± 0.57	1.27 ± 0.61
	Test	0.59 ± 0.22	0.57 ± 0.27

* Statistically significant difference between groups overtime (p<0.05)

## Discussion

This study investigated two different porcine-derived soft tissue grafting substitutes [CBM {Mucograft^®^ (Control)} and ADM {NovoMatrix^™^ (Test)}] as replacement grafts for the treatment of GR defects. While the employment of porcine-derived grafting materials is not new in the periodontal field,^21^ this is the first study, to the best of our knowledge, to histologically evaluate this porcine-derived ADM for the treatment of GR defects. Another grafting substitute, CBM, was selected as a control group for two main reasons: the common origin, and the extensively documented use of CBM in the literature in many periodontal plastic surgical procedures.^11^,^22^,^23^

Results from this investigation confirmed that both porcine-derived grafting materials are safe to use, demonstrating healthy soft tissues at the implantation bed at all time points. Tissue response was favorable, as both implanted materials were nonirritant and no signs of necrosis or persistent acute inflammatory reaction were observed. In addition, the grafting materials were well integrated without any foreign body reaction detected at any of the periods of time evaluated. Macroscopically, observations of all test and control sites also showed no significant differences at any interval and tissues did not show any signs of dehiscence, inflammation, or infection at 10 weeks.

The success of periodontal plastic surgery for the correction of GR defects relies primarily on two parameters: soft tissue coverage of the exposed root and tissue thickness. The coverage of the denuded root provides, the desired esthetic outcomes, and the increase in tissue thickness ensure long-term stability and prevents further recession. While lacking clinical measurements, this investigation quantitatively evaluated the histomorphometric results. Regarding tissue thickness, both grafting substitutes were able to successfully integrate with the surrounding tissues, demonstrating changes over the investigation period. Although the true increase in thickness could not be elucidated due to the lack of a negative control (CAF alone), the changes over time can be correlated with the remodeling of the soft tissue around the implanted materials. Both grafting materials suffered from reduction in their thickness at the coronal notch as well as a reduction of 2 mm bellow the gingival margin, which was more pronounced in the control group. The position of the gingival margin was also evaluated; at ten weeks, a total of 10 sections from control sites and 11 sections from test sites were appropriate for histomorphometric measurements. At this time point, 6 of the 10 control sections and 1 of the 11 test sections did not have coverage of the coronal notch, which meant that the distance from the notch to the gingival margin was reported as 0.0, and the buccolingual tissue thickness at the coronal notch was also reported as 0.0. At this point, the mean histomorphometric values tended to be higher for test than control sites, being statistically significantly different for the total mean of buccolingual tissue thickness and the distance from the gingival margin to the notch. As for the 6 weeks time point, and to account for the lack of measurements for these parameters, two means were reported for those values: one mean in which all values were included (including the 0.0 values) and one mean in which only positive values were included (Table 1). At the 2 weeks evaluation, all sections had coverage of the coronal notch, which meant that all measurements could be performed. Root coverage and buccolingual tissue thickness between sites treated with ADM and CBM were minimally different.

Despite several methodological differences and distinct methods for data interpretation, the outcomes of this investigation seem to agree with previous animal^24^ and human studies^9^,^10^,^25^ using ADM and CBM for the treatment of GR defects. Fickl, et al.^21^ investigated the use of a porcine dermal matrix in six patients and observed that, after 12 months, complete root coverage was only achieved in less than half of the defects. In addition, as occurred in this investigation, Fickl and colleagues found a reduction in root coverage over time. Similarly, Al-Hezaimi, et al.^24^ (2013), after performing CAF + ADM in dogs, observed the material markedly degraded over time, reaching levels similar to that of the control group without ADM. Results from this investigation also showed a decrease in thickness and coverage over time, possibly because of the resorption process of the grafting materials. Nevertheless, the utilization of the porcine-derived ADM as a soft tissue substitute provides substantial advantages by minimizing morbidity and reducing the surgical time.

This investigation exhibits several limitations. First, animals were analyzed up to 10 weeks. While a longer follow-up would have provided more meaningful clinical results, this study aimed to assess the healing response and tissue integration rather than elucidate the most effective material for soft tissue grafting procedures. Similarly, GR defects were created surgically and treated immediately after it. Hence, this scenario may not provide a reliable data to compare these materials with those of other animal studies. However, the histological data and the comparison amongst the two groups were possible although the lack of positive and negative control groups prevented further analysis. Once again, the purpose was to investigate the safety of and the tissue response to these grafting substitutes, which could be completed with the model employed. Future investigations should focus on the evaluation of the clinical outcomes of porcine ADM. Last, both materials can be applied in many clinical scenarios and combined with different surgical techniques, which should be also further explored. While the use of connective tissue graft has been repeatedly recognized as the gold standard, the employment of allo- and xenografting substitutes provide strategic advantages that would ensure the continuity of their clinical usage and investigations.

In conclusion, both grafting materials investigated can successfully integrate into the soft tissues and against the denuded root surface. Histological results revealed similar outcomes with successful integration and absence of adverse events. Histomorphometric outcomes were similar between groups at 2 weeks with increasing differences at 6 and 10 weeks. Test group provided greater root coverage and tissue thickness at the end of the follow-up period. Results from this study prompt the utilization of porcine-derived ADM for future clinical trials.
